# Crystal structure of *N*′-(2,6-di­methyl­phen­yl)benzene­carboximidamide tetra­hydro­furan monosolvate

**DOI:** 10.1107/S2056989014026255

**Published:** 2015-01-01

**Authors:** Jian-Ping Zhao, Rui-Qin Liu, Zhi-Hao Jiang, Sheng-Di Bai

**Affiliations:** a Institute of Applied Chemistry, Shanxi University, Taiyuan 030006, People’s Republic of China

**Keywords:** crystal structure, benzene­carboximidamide, tetra­hydro­furan solvate, hydrogen bonding

## Abstract

The asymmetric unit of the title compound, C_15_H_16_N_2_·C_4_H_8_O, contains two amidine mol­ecules (*A* and *B*) with slightly different conformations and two tetra­hydro­furan (THF) solvent mol­ecules. In the amidine mol­ecules, the di­methyl­phenyl ring and the NH_2_ group lie to the same side of the N=C bond and the dihedral angles between the aromatic rings are 54.25 (7) (mol­ecule *A*) and 58.88 (6) ° (mol­ecule *B*). In the crystal, N—H⋯N hydrogen bonds link the amidine mol­ecules into [100] *C*(4) chains of alternating *A* and *B* mol­ecules. Both amidine mol­ecules form an N—H⋯O hydrogen bond to an adjacent THF solvent mol­ecule.

## Related literature   

For reviews of related metal amidinates and their applications in ring-opening polymerization, see: Edelmann (1994 ▸); Bai *et al.* (2013 ▸); Qian *et al.* (2010 ▸); Bakthavachalam *et al.* (2014 ▸). For a related synthetic method for amidines, see: Liu *et al.* (2013 ▸). For a related crystal structure, see Zhang & Tong (2008 ▸).
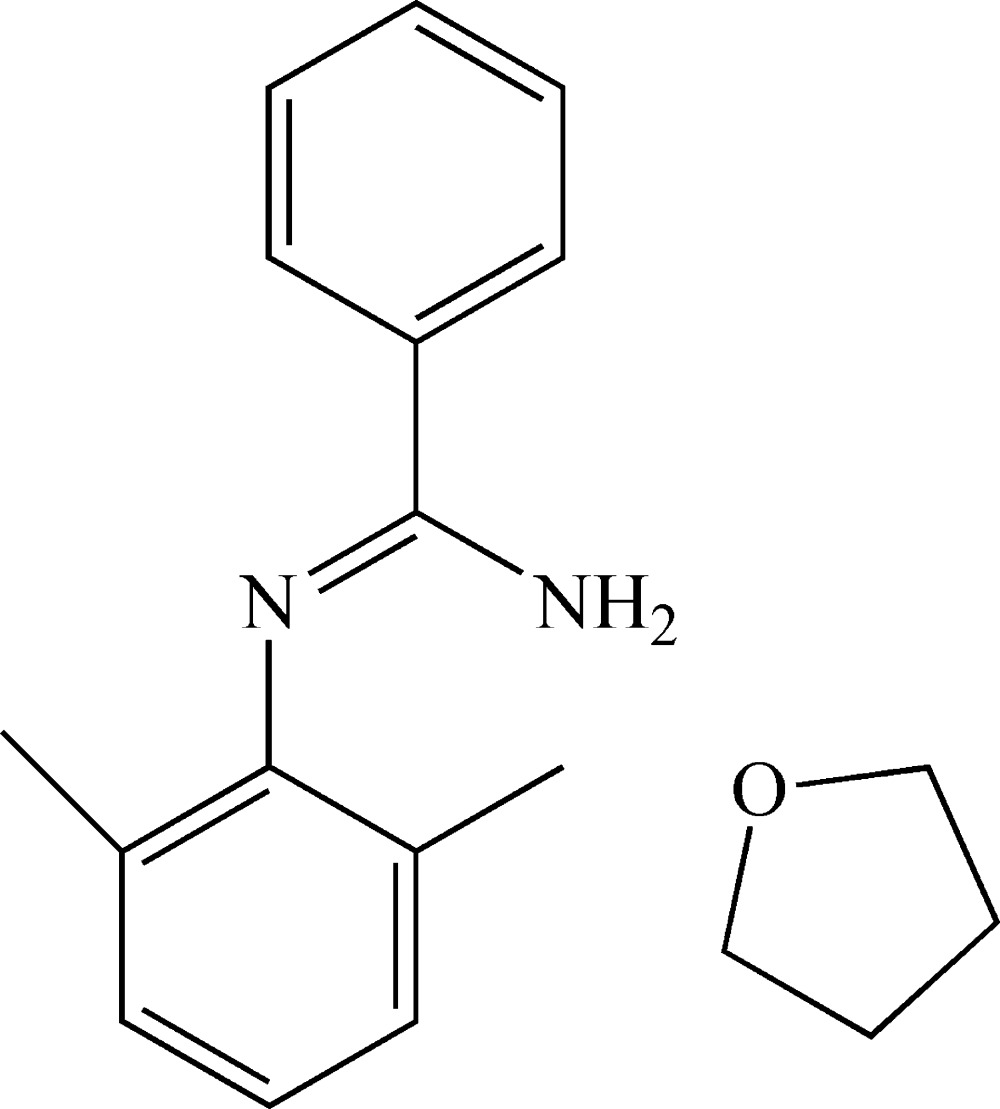


## Experimental   

### Crystal data  

C_15_H_16_N_2_·C_4_H_8_O
*M* *_r_* = 296.40
Monoclinic, 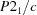

*a* = 10.075 (4) Å
*b* = 14.549 (6) Å
*c* = 24.208 (8) Å
β = 90.662 (8)°
*V* = 3548 (2) Å^3^
*Z* = 8
Mo *K*α radiation
μ = 0.07 mm^−1^
*T* = 200 K
0.30 × 0.30 × 0.25 mm

### Data collection  

Bruker SMART CCD diffractometer
Absorption correction: multi-scan (*SADABS*; Sheldrick, 1996 ▸) *T*_min_ = 0.980, *T*_max_ = 0.983
19431 measured reflections
6239 independent reflections
2958 reflections with *I* > 2σ(*I*)
*R*_int_ = 0.076

### Refinement  

*R*[*F* ^2^ > 2σ(*F* ^2^)] = 0.054
*wR*(*F* ^2^) = 0.159
*S* = 1.00
6239 reflections
402 parameters
1 restraint
H-atom parameters constrained
Δρ_max_ = 0.19 e Å^−3^
Δρ_min_ = −0.16 e Å^−3^

### 

Data collection: *SMART* (Bruker, 2000 ▸); cell refinement: *SAINT* (Bruker, 2000 ▸); data reduction: *SAINT*; program(s) used to solve structure: *SHELXS97* (Sheldrick, 2008 ▸); program(s) used to refine structure: *SHELXL97* (Sheldrick, 2008 ▸); molecular graphics: *SHELXTL/PC* (Sheldrick, 2008 ▸); software used to prepare material for publication: *SHELXL97*.

## Supplementary Material

Crystal structure: contains datablock(s) I, New_Global_Publ_Block. DOI: 10.1107/S2056989014026255/hb7332sup1.cif

Structure factors: contains datablock(s) I. DOI: 10.1107/S2056989014026255/hb7332Isup2.hkl

Supporting information file. DOI: 10.1107/S2056989014026255/hb7332Isup3.cml

**Fig. 1.**
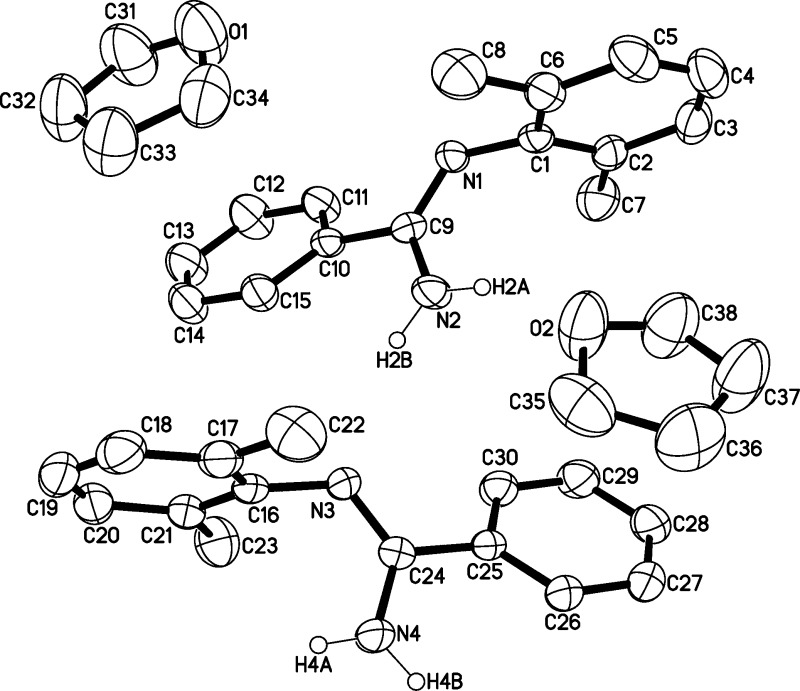
The asymmetric unit of the title compound, showing the atom-numbering scheme. Displacement ellipsoids were drawn at the 30% probability level. Hydrogen atoms, except for the nitrogen donor atoms, have been omitted for clarity.

. DOI: 10.1107/S2056989014026255/hb7332fig1.tif
The asymmetric unit of the title compound, showing the atom-numbering scheme. Displacement ellipsoids were drawn at the 30% probability level. Hydrogen atoms, except for the nitro­gen donor atoms, have been omitted for clarity.

**Fig. 2.**
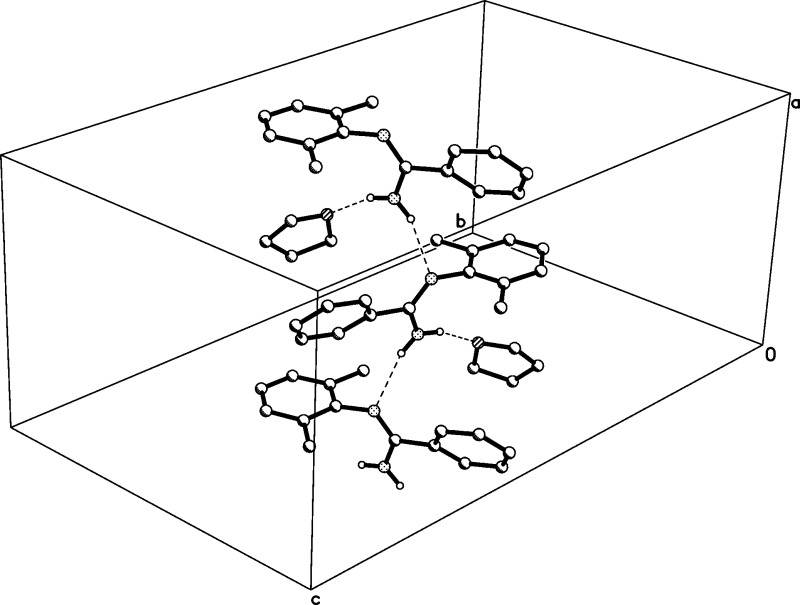
The view of one–dimensional chain in crystal structure of I. Symmetry codes: (i) x + 1, y, z.

I x y z . DOI: 10.1107/S2056989014026255/hb7332fig2.tif
The view of one–dimensional chain in crystal structure of **I**. Symmetry codes: (i) *x* + 1, *y*, *z*.

CCDC reference: 1036842

Additional supporting information:  crystallographic information; 3D view; checkCIF report

## Figures and Tables

**Table 1:** Hydrogen-bond geometry (, )

*D*H*A*	*D*H	H*A*	*D* *A*	*D*H*A*
N2H2*B*N3	0.88	2.27	3.123(3)	165
N4H4*B*N1^i^	0.88	2.22	3.061(3)	159
N2H2*A*O2	0.88	2.23	3.047(3)	155
N4H4*A*O1^i^	0.88	2.35	3.160(4)	153

Symmetry code: (i) 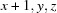.

## supplementary crystallographic information

### S1. Comment

Amidinate anions of the general formula [RC(NR')_2_]^-^ are the nitrogen analogs of the carboxylate anions. Their steric and electronic properties can be readily modified in a wide range through variation of the substituents on the carbon and nitrogen atoms. They have been widely employed as ligands in main group and transition metal coordination chemistry (Edelmann, 1994). Deprotonation of an amidine using a metal alkyl is a general synthetic method for preparing metal amidinato complexes, which could act as catalysts in ring-opening polymerization of lactones and lactides (Qian *et al.*, 2010; Bakthavachalam *et al.*, 2014). Herein we report the crystal structure of the title compound prepared by a one pot reaction with 2,6-dimethylaniline, LiBu*^n^*, PhCN and H_2_O.

The asymmetric unit of the title compound contains two amidines and two tetrahydronfuran molecules. Amidine molecules denoted *A* and *B* in the asymmetric unit possess different orientations. In molecule A, the phenyl ring C10—C15 and dimethylphenyl ring C1—C6 are twisted from the mean plane of N1/C9/N2 by 26.14 (18)° and 79.50 (8)°. Two N atoms connect the central C atom in different lengths of 1.293 (2) Å and 1.346 (2) Å, respectively. In molecule B, the phenyl ring C25—C30 and dimethylphenyl ring C16—C21 are twisted from the mean plane of N3/C24/N4 by 28.21 (18)° and 86.33 (8)°. Two N atoms connect the central C atom in different lengths of 1.288 (2) Å and 1.354 (2) Å. In the crystal, the intermolecular N—H···N hydrogen bonds link the molecules to give a one-dimension chain extending along the *a*-axis direction. The tetrahydrofuran molecules interact with the amidine chain *via* N—H···O hydrogen bonds. The compound is closely similar to the benzamidine with an *o*-tolyl substituent on the N atom, namely *N*^2^-*o*-Tolylbenzamidine (Zhang *et al.*, 2008), which has no tetrahydrofuran molecules attached.

### S2. Experimental

A solution of LiBu*^n^* (2.2 *M*, 2.27 ml, 5.0 mmol) in hexane was slowly added into a stirred solution of 2,6-dimethylaniline (0.62 ml, 5.0 mmol) in Et_2_O(*ca* 30 ml) by syringe at 273 K. The reaction mixture was warmed to room temperature and kept stirring for 3 h. Then benzonitrile (0.51 ml, 5.0 mmol) was added by syringe at 273 K. The reaction mixture was warmed to room temperature and kept stirring for 4 h. H_2_O (0.09 ml, 5.0 mmol) was added by syringe at 273 K. After stirred at room temperature for 4 h, the mixture was filtered and the filtrate was dried in vacuum to remove all volatiles. The residue was crystallized in hexane and gave colorless crystals, which was recrystallized from THF solution to give colorless blocks of the title compound (yield 1.17 g, 79%).^1^H NMR (300 MHz, CDCl_3_): δ = 7.97–6.91 (m, 8H; phenyl), 4.62 (s, 2H; N*H*_2_), 2.19 (s, 6; C*H*_3_). ^13^C NMR (75 MHz, CDCl_3_): δ = 135.8–122.9 (Ph), 18.0 (*C*H_3_).

### S3. Refinement

The methyl H atoms were constrained to an ideal geometry, with C—H distances of 0.98° and *U*_iso_(H) = 1.5*U*_eq_(C), but each group was allowed to rotate freely about its C—C bond. The methylene H atoms were constrained with C—H distances of 0.99° and *U*_iso_(H) = 1.2*U*_eq_(C). The phenyl H atoms were placed in geometrically idealized positions and constrained to ride on their parent atoms, with C—H distances of 0.95° and *U*_iso_(H) =1.2*U*_eq_(C).

### Crystal data

**Table:**

C_15_H_16_N_2_·C_4_H_8_O	*F*(000) = 1280
*M**_r_* = 296.40	*D*_x_ = 1.110 Mg m^−^^3^
Monoclinic, *P*2_1_/*c*	Mo *K*α radiation, λ = 0.71073 Å
*a* = 10.075 (4) Å	Cell parameters from 1838 reflections
*b* = 14.549 (6) Å	θ = 2.5–23.2°
*c* = 24.208 (8) Å	µ = 0.07 mm^−^^1^
β = 90.662 (8)°	*T* = 200 K
*V* = 3548 (2) Å^3^	Block, colorless
*Z* = 8	0.30 × 0.30 × 0.25 mm

### Data collection

**Table:**

Bruker SMART CCD diffractometer	6239 independent reflections
Radiation source: fine-focus sealed tube	2958 reflections with *I* > 2σ(*I*)
Graphite monochromator	*R*_int_ = 0.076
φ and ω scan	θ_max_ = 25.0°, θ_min_ = 1.6°
Absorption correction: multi-scan (*SADABS*; Sheldrick, 1996)	*h* = −11→11
*T*_min_ = 0.980, *T*_max_ = 0.983	*k* = −17→17
19431 measured reflections	*l* = −15→28

### Refinement

**Table:**

Refinement on *F*^2^	Secondary atom site location: difference Fourier map
Least-squares matrix: full	Hydrogen site location: inferred from neighbouring sites
*R*[*F*^2^ > 2σ(*F*^2^)] = 0.054	H-atom parameters constrained
*wR*(*F*^2^) = 0.159	*w* = 1/[σ^2^(*F*_o_^2^) + (0.0633*P*)^2^ + 0.1551*P*] where *P* = (*F*_o_^2^ + 2*F*_c_^2^)/3
*S* = 1.00	(Δ/σ)_max_ < 0.001
6239 reflections	Δρ_max_ = 0.19 e Å^−^^3^
402 parameters	Δρ_min_ = −0.16 e Å^−^^3^
1 restraint	Extinction correction: *SHELXL97* (Sheldrick, 2008), Fc^*^=kFc[1+0.001xFc^2^λ^3^/sin(2θ)]^-1/4^
Primary atom site location: structure-invariant direct methods	Extinction coefficient: 0.0052 (7)

### Special details

**Table:**

Geometry. All e.s.d.'s (except the e.s.d. in the dihedral angle between two l.s. planes) are estimated using the full covariance matrix. The cell e.s.d.'s are taken into account individually in the estimation of e.s.d.'s in distances, angles and torsion angles; correlations between e.s.d.'s in cell parameters are only used when they are defined by crystal symmetry. An approximate (isotropic) treatment of cell e.s.d.'s is used for estimating e.s.d.'s involving l.s. planes.
Refinement. Refinement of *F*^2^ against ALL reflections. The weighted *R*-factor *wR* and goodness of fit *S* are based on *F*^2^, conventional *R*-factors *R* are based on *F*, with *F* set to zero for negative *F*^2^. The threshold expression of *F*^2^ > σ(*F*^2^) is used only for calculating *R*-factors(gt) *etc*. and is not relevant to the choice of reflections for refinement. *R*-factors based on *F*^2^ are statistically about twice as large as those based on *F*, and *R*- factors based on ALL data will be even larger.

### Fractional atomic coordinates and isotropic or equivalent isotropic displacement parameters (Å^2^)

**Table:**

	*x*	*y*	*z*	*U*_iso_*/*U*_eq_	
N1	0.38595 (19)	0.73562 (14)	0.38856 (8)	0.0489 (6)	
N2	0.6122 (2)	0.69654 (16)	0.39181 (9)	0.0635 (7)	
H2A	0.6220	0.7139	0.4265	0.076*	
H2B	0.6803	0.6743	0.3737	0.076*	
N3	0.88493 (19)	0.64602 (14)	0.33988 (8)	0.0512 (6)	
N4	1.1129 (2)	0.65628 (15)	0.35904 (9)	0.0680 (7)	
H4A	1.1280	0.5992	0.3488	0.082*	
H4B	1.1788	0.6909	0.3709	0.082*	
C1	0.3862 (2)	0.77187 (18)	0.44302 (11)	0.0478 (7)	
C2	0.3850 (3)	0.86787 (19)	0.44921 (12)	0.0580 (7)	
C3	0.3632 (3)	0.9046 (2)	0.50151 (15)	0.0748 (9)	
H3	0.3627	0.9694	0.5062	0.090*	
C4	0.3425 (3)	0.8487 (3)	0.54637 (14)	0.0863 (11)	
H4	0.3248	0.8746	0.5815	0.104*	
C5	0.3475 (3)	0.7550 (3)	0.53989 (13)	0.0826 (10)	
H5	0.3354	0.7168	0.5712	0.099*	
C6	0.3699 (3)	0.7143 (2)	0.48870 (12)	0.0612 (8)	
C7	0.4045 (3)	0.9291 (2)	0.39988 (13)	0.0851 (10)	
H7A	0.4005	0.9936	0.4115	0.128*	
H7B	0.3345	0.9171	0.3724	0.128*	
H7C	0.4913	0.9165	0.3836	0.128*	
C8	0.3732 (3)	0.6115 (2)	0.48238 (13)	0.0922 (11)	
H8A	0.2986	0.5917	0.4589	0.138*	
H8B	0.3663	0.5826	0.5188	0.138*	
H8C	0.4568	0.5932	0.4653	0.138*	
C9	0.4927 (3)	0.70415 (17)	0.36643 (10)	0.0455 (6)	
C10	0.4851 (2)	0.67278 (17)	0.30779 (11)	0.0478 (7)	
C11	0.3895 (3)	0.7092 (2)	0.27270 (12)	0.0623 (8)	
H11	0.3302	0.7545	0.2861	0.075*	
C12	0.3790 (3)	0.6804 (2)	0.21812 (13)	0.0826 (10)	
H12	0.3125	0.7058	0.1945	0.099*	
C13	0.4645 (3)	0.6154 (3)	0.19833 (13)	0.0857 (10)	
H13	0.4579	0.5961	0.1609	0.103*	
C14	0.5598 (3)	0.5782 (2)	0.23253 (14)	0.0805 (10)	
H14	0.6188	0.5329	0.2189	0.097*	
C15	0.5697 (3)	0.6067 (2)	0.28703 (12)	0.0643 (8)	
H15	0.6357	0.5805	0.3105	0.077*	
C16	0.8975 (2)	0.55500 (18)	0.31850 (12)	0.0513 (7)	
C17	0.8837 (3)	0.4790 (2)	0.35303 (13)	0.0647 (8)	
C18	0.8782 (3)	0.3913 (2)	0.32926 (18)	0.0841 (10)	
H18	0.8693	0.3390	0.3525	0.101*	
C19	0.8853 (3)	0.3794 (2)	0.27344 (19)	0.0861 (11)	
H19	0.8800	0.3194	0.2581	0.103*	
C20	0.9001 (3)	0.4541 (2)	0.23951 (15)	0.0768 (9)	
H20	0.9051	0.4453	0.2007	0.092*	
C21	0.9080 (2)	0.5430 (2)	0.26115 (13)	0.0608 (8)	
C22	0.8773 (4)	0.4925 (2)	0.41468 (14)	0.1000 (11)	
H22A	0.8666	0.4327	0.4328	0.150*	
H22B	0.8016	0.5320	0.4235	0.150*	
H22C	0.9595	0.5215	0.4279	0.150*	
C23	0.9254 (4)	0.6242 (2)	0.22403 (12)	0.0913 (11)	
H23A	0.8530	0.6680	0.2299	0.137*	
H23B	0.9242	0.6039	0.1854	0.137*	
H23C	1.0105	0.6540	0.2324	0.137*	
C24	0.9884 (2)	0.69064 (17)	0.35657 (10)	0.0479 (6)	
C25	0.9695 (2)	0.78779 (17)	0.37466 (11)	0.0479 (7)	
C26	1.0508 (3)	0.82922 (19)	0.41377 (12)	0.0631 (8)	
H26	1.1229	0.7957	0.4294	0.076*	
C27	1.0285 (3)	0.9193 (2)	0.43048 (14)	0.0789 (9)	
H27	1.0849	0.9467	0.4575	0.095*	
C28	0.9254 (3)	0.9687 (2)	0.40811 (15)	0.0810 (10)	
H28	0.9104	1.0303	0.4195	0.097*	
C29	0.8436 (3)	0.9287 (2)	0.36907 (13)	0.0735 (9)	
H29	0.7716	0.9626	0.3536	0.088*	
C30	0.8659 (3)	0.83899 (19)	0.35218 (12)	0.0612 (8)	
H30	0.8096	0.8122	0.3249	0.073*	
C31	0.2488 (4)	0.4339 (4)	0.2957 (2)	0.1365 (17)	
H31A	0.2651	0.4871	0.2712	0.164*	
H31B	0.1603	0.4083	0.2863	0.164*	
C32	0.3484 (5)	0.3654 (3)	0.2872 (2)	0.1356 (17)	
H32A	0.3986	0.3784	0.2532	0.163*	
H32B	0.3077	0.3037	0.2839	0.163*	
C33	0.4359 (5)	0.3701 (3)	0.3359 (2)	0.1303 (15)	
H33A	0.5266	0.3888	0.3253	0.156*	
H33B	0.4406	0.3098	0.3547	0.156*	
C34	0.3747 (5)	0.4403 (3)	0.37231 (17)	0.1204 (14)	
H34A	0.3646	0.4156	0.4101	0.144*	
H34B	0.4313	0.4959	0.3743	0.144*	
C35	0.8550 (5)	0.6907 (3)	0.5224 (2)	0.1243 (15)	
H35A	0.9129	0.6905	0.4896	0.149*	
H35B	0.8520	0.6277	0.5378	0.149*	
C36	0.9064 (5)	0.7568 (4)	0.5648 (2)	0.1462 (18)	
H36A	0.9141	0.7269	0.6014	0.175*	
H36B	0.9945	0.7808	0.5543	0.175*	
C37	0.8081 (5)	0.8308 (3)	0.5658 (2)	0.1511 (19)	
H37A	0.7645	0.8332	0.6022	0.181*	
H37B	0.8511	0.8908	0.5589	0.181*	
C38	0.7154 (4)	0.8113 (3)	0.5243 (2)	0.1309 (16)	
H38A	0.6248	0.8226	0.5381	0.157*	
H38B	0.7302	0.8523	0.4923	0.157*	
O1	0.2509 (3)	0.4619 (2)	0.34960 (16)	0.1421 (12)	
O2	0.7271 (3)	0.7206 (2)	0.50790 (11)	0.1237 (10)	

### Atomic displacement parameters (Å^2^)

**Table:**

	*U*^11^	*U*^22^	*U*^33^	*U*^12^	*U*^13^	*U*^23^
N1	0.0377 (13)	0.0606 (14)	0.0482 (14)	0.0003 (10)	−0.0005 (10)	−0.0089 (11)
N2	0.0381 (14)	0.0985 (19)	0.0536 (15)	0.0037 (12)	−0.0051 (11)	−0.0178 (13)
N3	0.0396 (13)	0.0502 (13)	0.0638 (15)	0.0000 (10)	−0.0013 (11)	−0.0106 (11)
N4	0.0374 (14)	0.0586 (15)	0.1079 (19)	0.0003 (11)	−0.0044 (12)	−0.0200 (13)
C1	0.0341 (15)	0.0596 (18)	0.0498 (18)	0.0002 (12)	0.0003 (12)	−0.0029 (15)
C2	0.0462 (17)	0.062 (2)	0.066 (2)	0.0009 (13)	−0.0073 (14)	−0.0112 (16)
C3	0.064 (2)	0.078 (2)	0.082 (3)	0.0058 (16)	−0.0124 (18)	−0.029 (2)
C4	0.076 (2)	0.122 (3)	0.060 (2)	0.019 (2)	−0.0083 (18)	−0.029 (2)
C5	0.075 (2)	0.113 (3)	0.060 (2)	0.011 (2)	0.0049 (17)	0.003 (2)
C6	0.0533 (18)	0.074 (2)	0.056 (2)	0.0007 (15)	0.0007 (14)	−0.0007 (17)
C7	0.093 (3)	0.062 (2)	0.101 (3)	−0.0047 (17)	0.003 (2)	0.0069 (18)
C8	0.106 (3)	0.081 (3)	0.089 (3)	0.000 (2)	0.017 (2)	0.0199 (19)
C9	0.0378 (16)	0.0505 (16)	0.0482 (17)	−0.0052 (12)	−0.0005 (13)	0.0014 (12)
C10	0.0348 (15)	0.0565 (16)	0.0520 (18)	−0.0022 (12)	−0.0008 (13)	−0.0052 (14)
C11	0.0552 (19)	0.077 (2)	0.054 (2)	0.0126 (15)	−0.0001 (15)	−0.0026 (16)
C12	0.079 (2)	0.112 (3)	0.057 (2)	0.022 (2)	−0.0140 (17)	−0.0053 (19)
C13	0.075 (2)	0.125 (3)	0.057 (2)	0.009 (2)	−0.0085 (19)	−0.027 (2)
C14	0.059 (2)	0.108 (3)	0.074 (2)	0.0180 (18)	−0.0073 (18)	−0.037 (2)
C15	0.0450 (18)	0.082 (2)	0.066 (2)	0.0082 (15)	−0.0097 (15)	−0.0193 (16)
C16	0.0308 (15)	0.0516 (17)	0.071 (2)	−0.0002 (12)	0.0004 (13)	−0.0054 (15)
C17	0.0475 (18)	0.064 (2)	0.083 (2)	−0.0028 (14)	0.0045 (15)	0.0017 (18)
C18	0.065 (2)	0.058 (2)	0.129 (3)	−0.0055 (16)	0.001 (2)	0.003 (2)
C19	0.062 (2)	0.063 (2)	0.133 (4)	0.0011 (17)	−0.007 (2)	−0.024 (2)
C20	0.056 (2)	0.083 (3)	0.092 (3)	0.0080 (17)	−0.0096 (17)	−0.030 (2)
C21	0.0434 (17)	0.064 (2)	0.075 (2)	0.0049 (13)	−0.0011 (15)	−0.0091 (17)
C22	0.109 (3)	0.093 (3)	0.098 (3)	0.004 (2)	0.021 (2)	0.022 (2)
C23	0.111 (3)	0.091 (3)	0.072 (2)	0.001 (2)	0.004 (2)	−0.0036 (19)
C24	0.0383 (16)	0.0513 (16)	0.0542 (17)	−0.0001 (13)	0.0033 (13)	−0.0029 (13)
C25	0.0381 (15)	0.0484 (16)	0.0572 (18)	−0.0026 (13)	0.0051 (13)	−0.0027 (13)
C26	0.0473 (17)	0.064 (2)	0.078 (2)	0.0008 (14)	−0.0035 (15)	−0.0159 (16)
C27	0.058 (2)	0.076 (2)	0.103 (3)	−0.0021 (17)	−0.0027 (18)	−0.0324 (19)
C28	0.068 (2)	0.061 (2)	0.115 (3)	0.0013 (18)	0.016 (2)	−0.020 (2)
C29	0.061 (2)	0.059 (2)	0.101 (3)	0.0092 (16)	−0.0016 (18)	−0.0033 (18)
C30	0.0516 (18)	0.0562 (18)	0.076 (2)	0.0014 (15)	−0.0028 (15)	0.0004 (15)
C31	0.102 (4)	0.165 (5)	0.142 (4)	0.045 (3)	−0.016 (3)	−0.031 (4)
C32	0.114 (4)	0.128 (4)	0.164 (4)	0.035 (3)	−0.044 (3)	−0.059 (3)
C33	0.118 (4)	0.114 (4)	0.157 (4)	0.031 (3)	−0.034 (3)	−0.018 (3)
C34	0.147 (4)	0.105 (3)	0.109 (3)	−0.014 (3)	−0.006 (3)	−0.014 (3)
C35	0.129 (4)	0.114 (4)	0.131 (4)	0.030 (3)	0.026 (3)	0.011 (3)
C36	0.111 (4)	0.185 (5)	0.142 (4)	0.024 (4)	−0.057 (3)	−0.008 (4)
C37	0.139 (4)	0.130 (4)	0.182 (5)	0.015 (3)	−0.076 (4)	−0.044 (4)
C38	0.114 (4)	0.108 (4)	0.170 (4)	0.014 (3)	−0.054 (3)	−0.020 (3)
O1	0.126 (3)	0.144 (3)	0.156 (3)	0.042 (2)	−0.002 (2)	−0.048 (2)
O2	0.141 (3)	0.115 (2)	0.114 (2)	0.010 (2)	−0.0445 (19)	−0.0314 (17)

### Geometric parameters (Å, º)

**Table:**

N1—C9	1.291 (3)	C20—C21	1.397 (4)
N1—C1	1.420 (3)	C20—H20	0.9500
N2—C9	1.350 (3)	C21—C23	1.497 (4)
N2—H2A	0.8800	C22—H22A	0.9800
N2—H2B	0.8800	C22—H22B	0.9800
N3—C24	1.289 (3)	C22—H22C	0.9800
N3—C16	1.428 (3)	C23—H23A	0.9800
N4—C24	1.351 (3)	C23—H23B	0.9800
N4—H4A	0.8800	C23—H23C	0.9800
N4—H4B	0.8800	C24—C25	1.493 (3)
C1—C6	1.399 (4)	C25—C26	1.383 (3)
C1—C2	1.405 (4)	C25—C30	1.388 (3)
C2—C3	1.394 (4)	C26—C27	1.391 (4)
C2—C7	1.505 (4)	C26—H26	0.9500
C3—C4	1.374 (4)	C27—C28	1.369 (4)
C3—H3	0.9500	C27—H27	0.9500
C4—C5	1.373 (5)	C28—C29	1.376 (4)
C4—H4	0.9500	C28—H28	0.9500
C5—C6	1.394 (4)	C29—C30	1.387 (4)
C5—H5	0.9500	C29—H29	0.9500
C6—C8	1.504 (4)	C30—H30	0.9500
C7—H7A	0.9800	C31—O1	1.367 (4)
C7—H7B	0.9800	C31—C32	1.431 (5)
C7—H7C	0.9800	C31—H31A	0.9900
C8—H8A	0.9800	C31—H31B	0.9900
C8—H8B	0.9800	C32—C33	1.465 (5)
C8—H8C	0.9800	C32—H32A	0.9900
C9—C10	1.492 (3)	C32—H32B	0.9900
C10—C15	1.383 (3)	C33—C34	1.488 (5)
C10—C11	1.383 (3)	C33—H33A	0.9900
C11—C12	1.389 (4)	C33—H33B	0.9900
C11—H11	0.9500	C34—O1	1.393 (5)
C12—C13	1.370 (4)	C34—H34A	0.9900
C12—H12	0.9500	C34—H34B	0.9900
C13—C14	1.371 (4)	C35—O2	1.401 (5)
C13—H13	0.9500	C35—C36	1.496 (6)
C14—C15	1.386 (4)	C35—H35A	0.9900
C14—H14	0.9500	C35—H35B	0.9900
C15—H15	0.9500	C36—C37	1.462 (6)
C16—C17	1.394 (4)	C36—H36A	0.9900
C16—C21	1.404 (4)	C36—H36B	0.9900
C17—C18	1.400 (4)	C37—C38	1.393 (5)
C17—C22	1.507 (4)	C37—H37A	0.9900
C18—C19	1.365 (4)	C37—H37B	0.9900
C18—H18	0.9500	C38—O2	1.384 (4)
C19—C20	1.372 (4)	C38—H38A	0.9900
C19—H19	0.9500	C38—H38B	0.9900
			
C9—N1—C1	121.6 (2)	C17—C22—H22C	109.5
C9—N2—H2A	120.0	H22A—C22—H22C	109.5
C9—N2—H2B	120.0	H22B—C22—H22C	109.5
H2A—N2—H2B	120.0	C21—C23—H23A	109.5
C24—N3—C16	120.4 (2)	C21—C23—H23B	109.5
C24—N4—H4A	120.0	H23A—C23—H23B	109.5
C24—N4—H4B	120.0	C21—C23—H23C	109.5
H4A—N4—H4B	120.0	H23A—C23—H23C	109.5
C6—C1—C2	120.7 (3)	H23B—C23—H23C	109.5
C6—C1—N1	120.8 (2)	N3—C24—N4	125.1 (2)
C2—C1—N1	117.9 (2)	N3—C24—C25	117.6 (2)
C3—C2—C1	118.7 (3)	N4—C24—C25	117.3 (2)
C3—C2—C7	121.2 (3)	C26—C25—C30	118.1 (2)
C1—C2—C7	120.2 (3)	C26—C25—C24	122.5 (2)
C4—C3—C2	121.2 (3)	C30—C25—C24	119.4 (2)
C4—C3—H3	119.4	C25—C26—C27	120.9 (3)
C2—C3—H3	119.4	C25—C26—H26	119.6
C5—C4—C3	119.4 (3)	C27—C26—H26	119.6
C5—C4—H4	120.3	C28—C27—C26	120.3 (3)
C3—C4—H4	120.3	C28—C27—H27	119.9
C4—C5—C6	122.0 (3)	C26—C27—H27	119.9
C4—C5—H5	119.0	C27—C28—C29	119.7 (3)
C6—C5—H5	119.0	C27—C28—H28	120.2
C5—C6—C1	118.0 (3)	C29—C28—H28	120.2
C5—C6—C8	121.1 (3)	C28—C29—C30	120.2 (3)
C1—C6—C8	120.8 (3)	C28—C29—H29	119.9
C2—C7—H7A	109.5	C30—C29—H29	119.9
C2—C7—H7B	109.5	C29—C30—C25	120.9 (3)
H7A—C7—H7B	109.5	C29—C30—H30	119.6
C2—C7—H7C	109.5	C25—C30—H30	119.6
H7A—C7—H7C	109.5	O1—C31—C32	110.0 (4)
H7B—C7—H7C	109.5	O1—C31—H31A	109.7
C6—C8—H8A	109.5	C32—C31—H31A	109.7
C6—C8—H8B	109.5	O1—C31—H31B	109.7
H8A—C8—H8B	109.5	C32—C31—H31B	109.7
C6—C8—H8C	109.5	H31A—C31—H31B	108.2
H8A—C8—H8C	109.5	C31—C32—C33	105.6 (4)
H8B—C8—H8C	109.5	C31—C32—H32A	110.6
N1—C9—N2	125.7 (2)	C33—C32—H32A	110.6
N1—C9—C10	118.0 (2)	C31—C32—H32B	110.6
N2—C9—C10	116.3 (2)	C33—C32—H32B	110.6
C15—C10—C11	118.1 (2)	H32A—C32—H32B	108.8
C15—C10—C9	122.2 (2)	C32—C33—C34	105.0 (4)
C11—C10—C9	119.7 (2)	C32—C33—H33A	110.7
C10—C11—C12	120.9 (3)	C34—C33—H33A	110.7
C10—C11—H11	119.5	C32—C33—H33B	110.7
C12—C11—H11	119.5	C34—C33—H33B	110.7
C13—C12—C11	120.0 (3)	H33A—C33—H33B	108.8
C13—C12—H12	120.0	O1—C34—C33	107.2 (3)
C11—C12—H12	120.0	O1—C34—H34A	110.3
C12—C13—C14	120.0 (3)	C33—C34—H34A	110.3
C12—C13—H13	120.0	O1—C34—H34B	110.3
C14—C13—H13	120.0	C33—C34—H34B	110.3
C13—C14—C15	119.9 (3)	H34A—C34—H34B	108.5
C13—C14—H14	120.0	O2—C35—C36	106.4 (3)
C15—C14—H14	120.0	O2—C35—H35A	110.5
C10—C15—C14	121.1 (3)	C36—C35—H35A	110.5
C10—C15—H15	119.5	O2—C35—H35B	110.5
C14—C15—H15	119.5	C36—C35—H35B	110.5
C17—C16—C21	120.2 (3)	H35A—C35—H35B	108.6
C17—C16—N3	120.6 (3)	C37—C36—C35	104.9 (3)
C21—C16—N3	118.8 (2)	C37—C36—H36A	110.8
C16—C17—C18	118.7 (3)	C35—C36—H36A	110.8
C16—C17—C22	119.7 (3)	C37—C36—H36B	110.8
C18—C17—C22	121.6 (3)	C35—C36—H36B	110.8
C19—C18—C17	121.4 (3)	H36A—C36—H36B	108.9
C19—C18—H18	119.3	C38—C37—C36	106.6 (4)
C17—C18—H18	119.3	C38—C37—H37A	110.4
C18—C19—C20	120.0 (3)	C36—C37—H37A	110.4
C18—C19—H19	120.0	C38—C37—H37B	110.4
C20—C19—H19	120.0	C36—C37—H37B	110.4
C19—C20—C21	121.0 (3)	H37A—C37—H37B	108.6
C19—C20—H20	119.5	O2—C38—C37	110.0 (4)
C21—C20—H20	119.5	O2—C38—H38A	109.7
C20—C21—C16	118.8 (3)	C37—C38—H38A	109.7
C20—C21—C23	120.8 (3)	O2—C38—H38B	109.7
C16—C21—C23	120.4 (3)	C37—C38—H38B	109.7
C17—C22—H22A	109.5	H38A—C38—H38B	108.2
C17—C22—H22B	109.5	C31—O1—C34	108.2 (3)
H22A—C22—H22B	109.5	C38—O2—C35	107.8 (3)
			
C9—N1—C1—C6	−86.3 (3)	N3—C16—C17—C22	−9.8 (4)
C9—N1—C1—C2	102.3 (3)	C16—C17—C18—C19	−0.4 (4)
C6—C1—C2—C3	−1.9 (4)	C22—C17—C18—C19	−179.4 (3)
N1—C1—C2—C3	169.6 (2)	C17—C18—C19—C20	1.0 (5)
C6—C1—C2—C7	179.1 (2)	C18—C19—C20—C21	−0.1 (5)
N1—C1—C2—C7	−9.4 (4)	C19—C20—C21—C16	−1.4 (4)
C1—C2—C3—C4	−0.4 (4)	C19—C20—C21—C23	179.4 (3)
C7—C2—C3—C4	178.6 (3)	C17—C16—C21—C20	2.0 (4)
C2—C3—C4—C5	2.1 (5)	N3—C16—C21—C20	−170.5 (2)
C3—C4—C5—C6	−1.6 (5)	C17—C16—C21—C23	−178.8 (3)
C4—C5—C6—C1	−0.6 (4)	N3—C16—C21—C23	8.7 (4)
C4—C5—C6—C8	−179.1 (3)	C16—N3—C24—N4	−4.3 (4)
C2—C1—C6—C5	2.4 (4)	C16—N3—C24—C25	175.7 (2)
N1—C1—C6—C5	−168.8 (2)	N3—C24—C25—C26	151.3 (2)
C2—C1—C6—C8	−179.1 (3)	N4—C24—C25—C26	−28.6 (4)
N1—C1—C6—C8	9.7 (4)	N3—C24—C25—C30	−28.0 (3)
C1—N1—C9—N2	4.0 (4)	N4—C24—C25—C30	152.1 (2)
C1—N1—C9—C10	−176.2 (2)	C30—C25—C26—C27	0.7 (4)
N1—C9—C10—C15	−153.2 (2)	C24—C25—C26—C27	−178.6 (3)
N2—C9—C10—C15	26.6 (3)	C25—C26—C27—C28	−0.3 (5)
N1—C9—C10—C11	25.7 (3)	C26—C27—C28—C29	0.2 (5)
N2—C9—C10—C11	−154.5 (2)	C27—C28—C29—C30	−0.4 (5)
C15—C10—C11—C12	−0.2 (4)	C28—C29—C30—C25	0.8 (4)
C9—C10—C11—C12	−179.1 (3)	C26—C25—C30—C29	−0.9 (4)
C10—C11—C12—C13	−0.3 (5)	C24—C25—C30—C29	178.4 (2)
C11—C12—C13—C14	0.6 (5)	O1—C31—C32—C33	−14.6 (6)
C12—C13—C14—C15	−0.3 (5)	C31—C32—C33—C34	3.1 (5)
C11—C10—C15—C14	0.5 (4)	C32—C33—C34—O1	8.9 (5)
C9—C10—C15—C14	179.4 (3)	O2—C35—C36—C37	−8.4 (6)
C13—C14—C15—C10	−0.2 (5)	C35—C36—C37—C38	−4.4 (6)
C24—N3—C16—C17	93.1 (3)	C36—C37—C38—O2	16.2 (6)
C24—N3—C16—C21	−94.5 (3)	C32—C31—O1—C34	20.8 (6)
C21—C16—C17—C18	−1.1 (4)	C33—C34—O1—C31	−18.2 (5)
N3—C16—C17—C18	171.2 (2)	C37—C38—O2—C35	−22.1 (5)
C21—C16—C17—C22	177.9 (2)	C36—C35—O2—C38	18.3 (5)

### Hydrogen-bond geometry (Å, º)

**Table:**

*D*—H···*A*	*D*—H	H···*A*	*D*···*A*	*D*—H···*A*
N2—H2*B*···N3	0.88	2.27	3.123 (3)	165
N4—H4*B*···N1^i^	0.88	2.22	3.061 (3)	159
N2—H2*A*···O2	0.88	2.23	3.047 (3)	155
N4—H4*A*···O1^i^	0.88	2.35	3.160 (4)	153

Symmetry code: (i) *x*+1, *y*, *z*.
